# Atraumatic sacral fracture in a postpartum patient in the late postoperative period following lumbar decompression for disc herniation

**DOI:** 10.31744/einstein_journal/2025RC1630

**Published:** 2025-10-30

**Authors:** Edgar Santiago Valesin, Mariano Tamura Vieira Gomes, Guilherme Pianowski Pajanoti, Ariel Filipe Wuerges de Aquino, Mario Lenza, Luciano Miller Reis Rodrigues

**Affiliations:** 1 Hospital Israelita Albert Einstein São Paulo SP Brazil Hospital Israelita Albert Einstein, São Paulo, SP, Brazil.

**Keywords:** Sacrum, Lumbosacral region, Fracture, stresss, Postpartum period, Postoperative period, Pregnancy, Low back pain, Diagnosis, differential

## Abstract

Stress fractures of the sacrum are extremely rare differential diagnoses for persistent low back pain in pregnant and postpartum women. The common occurrence of low back pain due to mechanical overload on the spine, coupled with challenges in obtaining imaging studies during this phase of a woman's life, often delays and compromises the accurate diagnosis of sacral fatigue fractures. We report the rare case of a postpartum patient who underwent surgery for lumbar disc herniation one year and one month earlier and was diagnosed with an atraumatic sacral fracture.

## INTRODUCTION

Atraumatic sacral fractures are rare in postpartum women and can be classified as stress fractures when they occur in metabolically healthy women during pregnancy or early lactation. Two main types of atraumatic fractures occur under different pathophysiological conditions.^(1,2)^ Insufficiency fractures occur in patients with reduced bone mass, such as those with osteopenia or osteoporosis, who are typically elderly individuals subjected to routine biomechanical loads. Stress or fatigue fractures, on the other hand, affect athletes or amateurs engaged in endurance activities with prolonged impact, such as runners, without significant bone metabolism alterations. The primary symptom of a sacral stress fracture is low back pain radiating to the gluteal region. These nonspecific complaints contribute to the lesion being underdiagnosed and are often detected later.^(3)^

In the last trimester of pregnancy and in the immediate postpartum period, low back pain is a common complaint attributed primarily to weight gain, hormonal changes, imbalances in spinal and pelvic load distribution, and overload of the posterior musculoskeletal structures. These factors further hinder timely diagnosis.

This report highlights a rare cause of persistent low back pain in postpartum patients, discusses possible etiological factors, and outlines diagnostic challenges. The patient in this case also had a potential confounding factor, late postoperative status following L5-S1 microdecompression for disc herniation, which introduced an additional diagnostic bias due to overlapping symptoms.

## CASE REPORT

**Figure 1:**
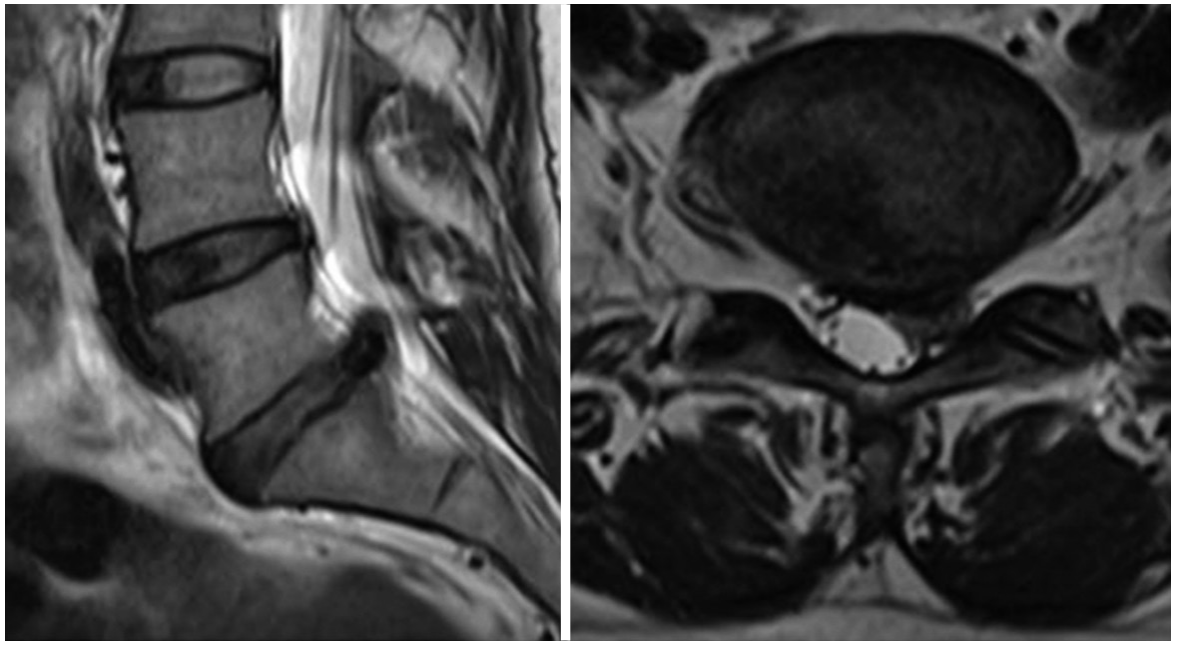
Preoperative MRI showing a left paramedian extruded disc herniation at L5-S1 compressing the dural sac and left S1 nerve root in the lateral recess (T2-weighted sagittal and axial views)

**Figure 2:**
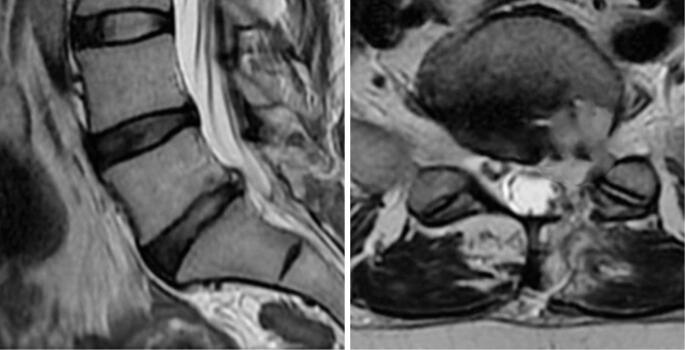
Postoperative MRI demonstrating microdecompression with left hemilaminotomy of L5 and flavectomy, along with edema in adjacent myoadipose planes and S1 root thickening. The previously identified extruded disc herniation compressing the nerve root is no longer present

A 46-year-old female patient with no comorbidities reported intense low back pain during the early postpartum period following her third spontaneous vaginal delivery in the absence of trauma. She had undergone orthopedic follow-up after lumbar discectomy one year and one month previously, which resulted in complete resolution of her preoperative low back pain and left-sided sciatica (Figures 1 and 2).

Postoperatively, the patient completed 20 sessions of physical therapy and returned to her residence abroad, where she maintained a light walking routine without engaging in additional physical activities. At that time, she weighed approximately 7kg, which was above her ideal body weight.

Approximately three months after the spinal surgery, she became pregnant for the third time. During pregnancy, the patient gained an additional 12kg in weight. The obstetric care and prenatal laboratory assessments were uneventful. The patient underwent spontaneous vaginal delivery without complications, with the total labor lasting approximately four hours.

In the first postpartum week, she experienced severe right-sided lumbosacral pain, which was progressive in intensity, without any history of falls, trauma, or specific mechanical triggers. The pain persisted for weeks, and the patient was unresponsive to analgesics and conservative measures. At eight weeks postpartum, an orthopedic evaluation was performed because of the persistent symptoms.

Physical examination revealed significant right-sided paravertebral and sacrococcygeal muscle spasms with tenderness on deep palpation in the lower lumbar, sacral, and coccygeal regions. The patient's gait was antalgic with a mild right-sided limp and worsening pain during an ipsilateral single-leg stance. Discrete compensatory antalgic scoliosis was also observed. The lumbar spine range of motion was partially limited, and specific provocative maneuvers, including the Lasègue, Gaenslen, and Patrick-Fabere tests, were negative. No sensory or motor deficits or signs of radiculopathy or myelopathy were observed.

**Figure 3:**
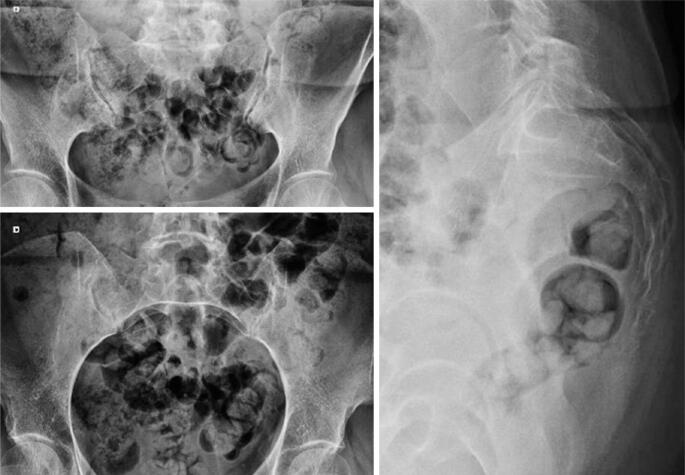
Radiograph of the pelvis and lumbar spine obtained during postpartum orthopedic evaluation. No evident signs of fracture were observed, despite persistent localized lumbosacral pain

**Figure 4:**
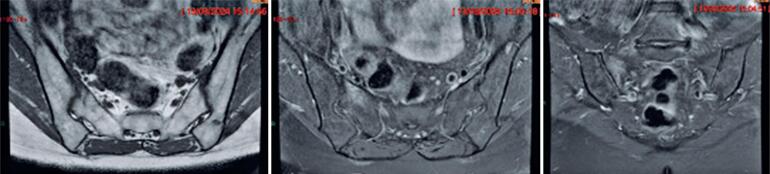
Axial T1, axial T2 with fat suppression, and coronal T2 with fat suppression MRI images showing an incomplete vertical fracture line without displacement on the anterior aspect of the right sacral ala, surrounded by mild bone edema, indicative of a subacute stress reaction

Plain radiographs of the lumbopelvic region (Figure 3) did not show clear evidence of fracture or instability. However, magnetic resonance imaging (MRI) revealed a right sacral fracture (Figure 4). Computed tomography was considered, but not performed, as the MRI findings were deemed sufficient for diagnosis and management.

Conservative treatment, including rest and physiotherapy, was initiated, leading to gradual improvement in pain and functional limitations. The clinical picture was consistent with an atraumatic sacral insufficiency fracture, likely associated with postpartum biomechanical stress and prior spinal surgery, despite the absence of classical risk factors, such as osteoporosis or corticosteroid use.

This case study was approved by the Research Ethics Committee of *Hospital Israelita Albert Einstein* (CAAE: 85432024.9.0000.0071; # 7.312.165), and written informed consent was obtained from the patient.

## DISCUSSION

Low back pain is one of the most frequent diagnoses in clinical practice and is the leading reason for adult emergency orthopedic care.^(4)^ This complaint is more prevalent during pregnancy. Studies have indicated that its incidence increases as pregnancy progresses, affecting approximately 47% of women in their third trimester.^(5)^ In the immediate postpartum period, the incidence rates increase further, with up to 67% of women reporting low back pain. During outpatient follow-up, the rate decreases to 37%, with most patients experiencing a gradual resolution of symptoms.^(6)^ Additionally, women with preexisting low back pain have a twofold increased risk of developing low back pain during pregnancy.^(7,8)^

Given the prevalence of low back pain in late pregnancy and the early postpartum period, sacral fractures present a diagnostic challenge for healthcare providers. In the present case, the diagnosis was further complicated by the patient's recent lumbar spine surgery.

Atraumatic sacral fractures may result from repetitive overload on healthy bone (stress) or normal loads on vulnerable bones (insufficiency). Sacral fractures during pregnancy and the postpartum period are likely a combination of these mechanisms. The literature suggests that distinguishing between the two can be challenging because of transient osteopenia associated with the high metabolic demand of pregnancy and lactation, which weakens the bone tissue, while biomechanical overload impacts the lumbar and pelvic regions.^(9–11)^ Maternal age, excessive weight gain, nutritional deficiencies, sarcopenia, labor conditions, and number of previous deliveries are all potential risk factors.

From a pathophysiological perspective, pregnancy induces a cascade of hormonal and biomechanical changes that increase vulnerability to sacral and pelvic insufficiency fractures. Hormones such as relaxin and progesterone lead to ligamentous laxity, particularly in the sacroiliac joints and pelvic ring, aiming to facilitate childbirth; however, they reduce local joint stability. Additionally, the progression of lumbar hyperlordosis and anterior pelvic tilt throughout gestation leads to a shift in the body's center of gravity, increasing the axial and shear forces transmitted to the lumbosacral junction and sacral ala, especially in individuals with altered biomechanics due to previous spinal surgeries.^(9–11)^

The postpartum period is marked by a high calcium demand for lactation, which, when combined with relative inactivity or nutritional compromise, can exacerbate transient bone demineralization.^(11)^ Although most women maintain calcium homeostasis through increased intestinal absorption, a subset may experience microarchitectural bone changes, particularly in trabecular-rich areas, such as the sacrum.

In the present case, laboratory evaluations of serum calcium, ionized calcium, phosphorus, and vitamin D levels were normal. However, the patient had an advanced maternal age (46 years), had gained 12kg during pregnancy, and had recently undergone lumbar spine surgery, which led to prolonged physical inactivity and progressive weight gain.

Biomechanical factors during labor, particularly the lithotomy position with hip hyperflexion and sacral pressure, may further stress the sacral bone.^(12)^ Although our patient did not report any specific traumatic events during delivery, the cumulative effects of these physiological changes and physical stressors may have contributed to the development of the insufficiency fracture.^(13)^

The clinical course of atraumatic sacral fractures in pregnant and postpartum patients is generally favorable with conservative treatment, as described in a few case reports. This includes relative rest for at least 6 weeks,^(13)^ physiotherapy, and nutritional support. Surgical interventions, such as bone cement augmentation analogous to vertebroplasty with polymethylmethacrylate, have been proposed for refractory cases; however, they lack robust scientific evidence.^(1)^ Currently, no standardized treatment protocol exists, and various conservative approaches, including prolonged rest, hydrotherapy, electromagnetic therapy, ultrasound, shockwave therapy, and pharmacological options (e.g., bisphosphonates, PTH, calcitonin, and raloxifene), have been utilized.

In this case, the patient experienced progressive improvement through physiotherapy, relative load protection, and the necessary postpartum adaptations to care for the newborn.

## FUTURE QUESTIONS

Unanswered questions remain for future research: What is the prevalence of pregnancy-related sacral fractures and how are they related to common low back pain during this phase? What is the impact of prior spinal surgeries in this context? What duration and type of rehabilitation are safe after spinal surgery to minimize skeletal overload risk during subsequent pregnancies? Should patients with persistent or worsening low back pain during pregnancy or the postpartum period be promptly evaluated with MRI, regardless of previous spinal conditions?

## CONCLUSION

Sacral stress fracture is a rare but important differential diagnosis for persistent low back and gluteal pain in late pregnancy and the early postpartum period. Although the exact pathophysiology remains unclear, the interplay between the mother's high metabolic demands and mechanical overload in the lumbosacral region likely contributes to this multifactorial and atraumatic lesion. Diagnosing these fractures is particularly challenging in patients with preexisting spinal conditions. Further studies are needed to clarify the risk factors and determine which patients should undergo imaging studies during pregnancy and the postpartum period.

## References

[1] Longhino V, Bonora C, Sansone V (2011). The management of sacral stress fractures: current concepts. Clin Cases Miner Bone Metab.

[2] Vajapey S, Matic G, Hartz C, Miller TL (2019). Sacral stress fractures: a rare but curable cause of back pain in athletes. Sports Health.

[3] Rodrigues LM, Ueno FH, Valesin ES, Fujiki EN, Milani C (2009). Sacral stress fracture in a runner: a case report. Clinics (Sao Paulo).

[4] Gotfryd AO, Valesin ES, Viola DC, Lenza M, da Silva JA, Emi AS (2015). Análise epidemiológica, de hábitos de vida e de fatores psicossociais de pacientes com dorsolombalgia em unidade de pronto atendimento ortopédico. einstein (Sao Paulo).

[5] Salari N, Mohammadi A, Hemmati M, Hasheminezhad R, Kani S, Shohaimi S (2023). The global prevalence of low back pain in pregnancy: a comprehensive systematic review and meta-analysis. BMC Pregnancy Childbirth.

[6] Ostgaard HC, Andersson GB (1992). Postpartum low-back pain. Spine.

[7] Saxena AK, Chilkoti GT, Singh A, Yadav G (2019). Pregnancy-induced low back pain in Indian women: Prevalence, risk factors, and correlation with serum calcium levels. Anesth Essays Res.

[8] Heuch I, Hagen K, Storheim K, Zwart J-A (2020). Associations between the number of children, age at childbirths and prevalence of chronic low back pain: the Nord-Trøndelag Health Study. BMC Public Health.

[9] Schmid L, Pfirrmann C, Hess T, Schlumpf U (1999). Bilateral fracture of the sacrum associated with pregnancy: a case report. Osteoporos Int.

[10] Dussa CU, El Daief SG, Sharma SD, Hughes PL (2005). Atraumatic fracture of the sacrum in pregnancy. J Obstet Gynaecol.

[11] De Búrca N (2012). Low back pain post partum - a case report. Man Ther.

[12] Deschamps Perdomo A, Tomé-Bermejo F, Piñera AR, Alvarez L (2015). Misdiagnosis of sacral stress fracture: an underestimated cause of low back pain in pregnancy?. Am J Case Rep.

[13] Giannoulis DK, Koulouvaris P, Zilakou E, Papadopoulos DB, Lykissas MG, Mavrodontidis AN (2015). Atraumatic Sacral Fracture in Late Pregnancy: A Case Report. Global Spine J.

